# Supplementary material for: Balance between breadth and depth in human many-alternative decisions
Source: eLife. 2022 Sep 15;11:e76985. doi: 10.7554/eLife.76985 (PMC9578699; doi:10.7554/eLife.76985)
Supplement: Supplementary file 1. [file elife-76985-supp1.docx]

| environment  capacity | poor | neutral | rich |
| --- | --- | --- | --- |
| 2 | $\boldsymbol{V}_{\boldsymbol{66}}\boldsymbol{=231.0}$  $\boldsymbol{p}_{\boldsymbol{adj}}\boldsymbol{=1.56}\boldsymbol{\times10}^{\boldsymbol{-3}}$ | $\boldsymbol{V}_{\boldsymbol{66}}\boldsymbol{=276.0}$  $\boldsymbol{p}_{\boldsymbol{adj}}\boldsymbol{=7.39\times}\boldsymbol{10}^{\boldsymbol{-4}}$ | $\boldsymbol{V}_{\boldsymbol{66}}\boldsymbol{=171.0}$  $\boldsymbol{p}_{\boldsymbol{adj}}\boldsymbol{=5.97\times}\boldsymbol{10}^{\boldsymbol{-3}}$ |
| 3 | $\boldsymbol{V}_{\boldsymbol{33}}\boldsymbol{=190.0}$  $\boldsymbol{p}_{\boldsymbol{adj}}\boldsymbol{=3.99}\boldsymbol{\times10}^{\boldsymbol{-3}}$ | $\boldsymbol{V}_{\boldsymbol{33}}\boldsymbol{=435.0}$  $\boldsymbol{p}_{\boldsymbol{adj}}\boldsymbol{=8.38\times}\boldsymbol{10}^{\boldsymbol{-5}}$ | $\boldsymbol{V}_{\boldsymbol{33}}\boldsymbol{=19.5}$  $\boldsymbol{p}_{\boldsymbol{adj}}\boldsymbol{=3.80\times}\boldsymbol{10}^{\boldsymbol{-4}}$ |
| 4 | $\boldsymbol{V}_{\boldsymbol{66}}\boldsymbol{=1128.0}$  $\boldsymbol{p}_{\boldsymbol{adj}}\boldsymbol{=7.89\times}\boldsymbol{10}^{\boldsymbol{-8}}$ | $V_{66}=1075.5$  $p_{adj}=1$ | $V_{66}=1196.0$  $p_{adj}=1$ |
| 5 | $\boldsymbol{V}_{\boldsymbol{33}}\boldsymbol{=435.0}$  $\boldsymbol{p}_{\boldsymbol{adj}}\boldsymbol{=8.65\times}\boldsymbol{10}^{\boldsymbol{-5}}$ | $\boldsymbol{V}_{\boldsymbol{33}}\boldsymbol{=410.0}$  $\boldsymbol{p}_{\boldsymbol{adj}}\boldsymbol{=.00881}$ | $V_{33}=152.0$  $p_{adj}=1$ |
| 6 | $\boldsymbol{V}_{\boldsymbol{33}}\boldsymbol{=300.0}$  $\boldsymbol{p}_{\boldsymbol{adj}}\boldsymbol{=6.10\times}\boldsymbol{10}^{\boldsymbol{-4}}$ | $V_{33}=331.5$  $p_{adj}=1$ | $V_{33}=134.0$  $p_{adj}=1$ |
| 7 | $\boldsymbol{V}_{\boldsymbol{33}}\boldsymbol{=473.0}$  $\boldsymbol{p}_{\boldsymbol{adj}}\boldsymbol{=.0197}$ | $\boldsymbol{V}_{\boldsymbol{33}}\boldsymbol{=443.5}$  $\boldsymbol{p}_{\boldsymbol{adj}}\boldsymbol{=.0268}$ | $V_{33}=267.5$  $p_{adj}=1$ |
| 8 | $\boldsymbol{V}_{\boldsymbol{66}}\boldsymbol{=1930.0}$  $\boldsymbol{p}_{\boldsymbol{adj}}\boldsymbol{=6.96\times}\boldsymbol{10}^{\boldsymbol{-7}}$ | $V_{66}=1424.0$  $p_{adj}=.340$ | $V_{66}=808.5$  $p_{adj}=1$ |
| 9 | $V_{33}=419.5$  $p_{adj}=.123$ | $V_{33}=287.0$  $p_{adj}=1$ | $V_{33}=199.0$  $p_{adj}=1$ |
| 10 | $V_{33}=428.0$  $p_{adj}=.0736$ | $V_{33}=420.5$  $p_{adj}=.417$ | $V_{33}=118.0$  $p_{adj}=.366$ |
| 16 | $V_{33}=365.0$  $p_{adj}=1$ | $V_{33}=236.0$  $p_{adj}=1$ | $\boldsymbol{V}_{\boldsymbol{33}}\boldsymbol{=64.5}$  $\boldsymbol{p}_{\boldsymbol{adj}}\boldsymbol{=6.53\times}\boldsymbol{10}^{\boldsymbol{-3}}$ |
| 32 | $V_{33}=266.0$  $p_{adj}=1$ | $V_{33}=43.0$  $p_{adj}=7.52\times{10}^{-4}$ | $\boldsymbol{V}_{\boldsymbol{33}}\boldsymbol{=28.0}$  $\boldsymbol{p}_{\boldsymbol{adj}}\boldsymbol{=2.21\times}\boldsymbol{10}^{\boldsymbol{-4}}$ |

***Tabl***$\boldsymbol{e}$ ***S1.*** Participants’ sampling strategy deviates from optimality and tends to be tilted toward depth at low capacity and breadth at high capacity. Results of one-sample Wilcoxon tests against the null hypothesis ($\mu$=0, no bias towards depth nor depth) of the differences between the optimal ($M_{opt}$) and the observed ($M$) number of alternatives sampled averaged per participant for each capacity and environment. $V$ statistics and p-values adjusted with Bonferroni corrections for multiple comparisons are reported and significant results ($\alpha$<.05) are highlighted in bold.
