# Supplementary material for: Balance between breadth and depth in human many-alternative decisions
Source: eLife. 2022 Sep 15;11:e76985. doi: 10.7554/eLife.76985 (PMC9578699; doi:10.7554/eLife.76985)
Supplement: Supplementary file 2. [file elife-76985-supp2.docx]

| block’s history  environment | first or alone | after poor | after neutral | after rich |
| --- | --- | --- | --- | --- |
| poor | $V=271,$  $p_{adj}=.070$ |  | $t=-3.19,$  $p_{adj}=.052$ | $t=0.026,$  $p_{adj}=1$ |
| neutral | $t=0.86,$  $p_{adj}=1$ | $t=1.73,$  $p_{adj}=.67$ |  | $t=0.25,$  $p_{adj}=1$ |
| rich | $\boldsymbol{V=693,}$  $\boldsymbol{p}_{\boldsymbol{adj}}\boldsymbol{=.0061}$ | $t=0.72,$  $p_{adj}=1$ | $V=49,$  $p_{adj}=1$ |  |

***Table S2***. Post-hoc comparisons (paired t-tests or Wilcoxon tests) between deviations from optimality in power factors $a$ extracted from fitting the power law model to BD trade-offs in first and second halves of blocks separately. Comparisons are performed inside each environment condition and depending on whether the block is presented alone (between-subjects designs) or first (within-subjects designs) or after another environment (within-subjects designs). P-values are corrected for multiple-comparisons using Bonferroni corrections and significant results (*p*<.05) are highlighted in bold.
