# Supplementary material for: Balance between breadth and depth in human many-alternative decisions
Source: eLife. 2022 Sep 15;11:e76985. doi: 10.7554/eLife.76985 (PMC9578699; doi:10.7554/eLife.76985)
Supplement: Supplementary file 3. [file elife-76985-supp3.docx]

| block’s history  block’s half | first or alone | after poor | after neutral | after rich |
| --- | --- | --- | --- | --- |
| first | $t=-0.40,$  $p_{adj}=1$ | $\boldsymbol{t=3.53,}$  $\boldsymbol{p}_{\boldsymbol{adj}}\boldsymbol{=.005}$ | $t=-1.31,$  $p_{adj}=.82$ | $\boldsymbol{t=-2.92,}$  $\boldsymbol{p}_{\boldsymbol{adj}}\boldsymbol{=.031}$ |
| second | $t=-1.16,$  $p_{adj}=1$ | $\boldsymbol{t=2.88,}$  $\boldsymbol{p}_{\boldsymbol{adj}}\boldsymbol{=.034}$ | $t=-0.80,$  $p_{adj}=1$ | $t=-2.61,$  $p_{adj}=.063$ |

***Table S3***. Post-hoc analyses (one sample t-tests with $mu=0$) of the deviations from optimality in power factors $a$ extracted from fitting the power law model to BD trade-offs in first and second halves of blocks separately. Comparisons are performed inside each block’s half (median split on the total number of trials) and depending on whether the block is presented alone (between-subjects designs) or first (within-subjects designs) or after another environment: poor, neutral or rich (within-subjects designs). P-values are corrected for multiple-comparisons using Bonferroni corrections and significant results (*p*<.05) are highlighted in bold.
