# Supplementary material for: Balance between breadth and depth in human many-alternative decisions
Source: eLife. 2022 Sep 15;11:e76985. doi: 10.7554/eLife.76985 (PMC9578699; doi:10.7554/eLife.76985)
Supplement: Supplementary file 5. [file elife-76985-supp5.docx]

|  | depth | square root | linear | optimal | power |
| --- | --- | --- | --- | --- | --- |
| pure breadth | $V_{126}=3748,$  $p_{adj}=1$ | $V_{126}=3582,$  $p_{adj}=1$ | $\boldsymbol{V}_{\boldsymbol{126}}\boldsymbol{=1443,}$  $\boldsymbol{p}_{\boldsymbol{adj}}\boldsymbol{=7.32}\boldsymbol{\times10}^{\boldsymbol{-4}}$ | $\boldsymbol{V}_{\boldsymbol{126}}\boldsymbol{=991,}$  $\boldsymbol{p}_{\boldsymbol{adj}}\boldsymbol{=3.56}\boldsymbol{\times10}^{\boldsymbol{-12}}$ | $\boldsymbol{V}_{\boldsymbol{126}}\boldsymbol{=802,}$  $\boldsymbol{p}_{\boldsymbol{adj}}\boldsymbol{=5.31}\boldsymbol{\times10}^{\boldsymbol{-12}}$ |
| depth |  | $V_{126}=4039,$  $p_{adj}=1$ | $\boldsymbol{V}_{\boldsymbol{126}}\boldsymbol{=2501,}$  $\boldsymbol{p}_{\boldsymbol{adj}}\boldsymbol{=.004}$ | $\boldsymbol{V}_{\boldsymbol{126}}\boldsymbol{=1252,}$  $\boldsymbol{p}_{\boldsymbol{adj}}\boldsymbol{=3.33}\boldsymbol{\times10}^{\boldsymbol{-10}}$ | $\boldsymbol{V}_{\boldsymbol{126}}\boldsymbol{=1025,}$  $\boldsymbol{p}_{\boldsymbol{adj}}\boldsymbol{=6.56}\boldsymbol{\times10}^{\boldsymbol{-12}}$ |
| square root |  |  | $V_{126}=2910,$  $p_{adj}=.119$ | $\boldsymbol{V}_{\boldsymbol{126}}\boldsymbol{=2347,}$  $\boldsymbol{p}_{\boldsymbol{adj}}\boldsymbol{=8.54}\boldsymbol{\times10}^{\boldsymbol{-4}}$ | $\boldsymbol{V}_{\boldsymbol{126}}\boldsymbol{=799,}$  $\boldsymbol{p}_{\boldsymbol{adj}}\boldsymbol{=4.23}\boldsymbol{\times10}^{\boldsymbol{-13}}$ |
| linear |  |  |  | $V_{126}=3485,$  $p_{adj}=1$ | $\boldsymbol{V}_{\boldsymbol{126}}\boldsymbol{=480,}$  $\boldsymbol{p}_{\boldsymbol{adj}}\boldsymbol{=6.08}\boldsymbol{\times10}^{\boldsymbol{-15}}$ |
| optimal |  |  |  |  | $\boldsymbol{V}_{\boldsymbol{126}}\boldsymbol{=2630,}$  $\boldsymbol{p}_{\boldsymbol{adj}}\boldsymbol{=.013}$ |

***Table S5***. Summary of the pair-wise comparisons (Wilcoxon Matched Pairs Signed-Ranks test) of the 4-folds averaged CVLL between all six models using Gaussian distributed noise. P-values are adjusted with Bonferroni corrections and significative differences (*p* <.05) are highlighted in bold. Models are ordered from worst (pure breadth) to best (free power-law).
