# Supplementary material for: Balance between breadth and depth in human many-alternative decisions
Source: eLife. 2022 Sep 15;11:e76985. doi: 10.7554/eLife.76985 (PMC9578699; doi:10.7554/eLife.76985)
Supplement: Supplementary file 6. [file elife-76985-supp6.docx]

| Designs  tests | B10 | W10 | B32 | W32 |
| --- | --- | --- | --- | --- |
| ANOVA | $\boldsymbol{K-W \chi}_{\boldsymbol{2}}^{\boldsymbol{2}}\boldsymbol{=16.65,}$  $\boldsymbol{p=2.43}\boldsymbol{\times10}^{\boldsymbol{-4}}$ | $\boldsymbol{F}_{\boldsymbol{1}}\boldsymbol{=23.21,}$  $\boldsymbol{p=1.61}\boldsymbol{\times10}^{\boldsymbol{-4}}$ | $F_{1}=1.62,$  $p=.21$ | $\boldsymbol{F}_{\boldsymbol{1}}\boldsymbol{=8.68,}$  $\boldsymbol{p=.009}$ |
| Poor vs. Neutral | $\boldsymbol{W}_{\boldsymbol{15}}\boldsymbol{=206,}$  $\boldsymbol{p}_{\boldsymbol{adj}}\boldsymbol{=3.63}\boldsymbol{\times10}^{\boldsymbol{-4}}$ | $\boldsymbol{t}_{\boldsymbol{17}}\boldsymbol{=3.81,}$  $\boldsymbol{p}_{\boldsymbol{adj}}\boldsymbol{=.004}$ | - | $t_{17}=1.37,$  $p_{adj}=.56$ |
| Poor vs. Rich | $\boldsymbol{W}_{\boldsymbol{15}}\boldsymbol{=188,}$  $\boldsymbol{p}_{\boldsymbol{adj}}\boldsymbol{=.005}$ | $\boldsymbol{t}_{\boldsymbol{17}}\boldsymbol{=4.82,}$  $\boldsymbol{p}_{\boldsymbol{adj}}\boldsymbol{=4.83}\boldsymbol{\times10}^{\boldsymbol{-4}}$ | - | $\boldsymbol{t}_{\boldsymbol{17}}\boldsymbol{=2.95,}$  $\boldsymbol{p}_{\boldsymbol{adj}}\boldsymbol{=.027}$ |
| Neutral vs. Rich | $W_{15}=126,$  $p_{adj}=1$ | $t_{17}=0.42,$  $p_{adj}=1$ | - | $t_{17}=2.32,$  $p_{adj}=.10$ |

***Table S6***. Summary of the comparisons between the averaged individual 4-fold CVLL in each environment and experimental design using Gaussian distributed noise. In the first row are displays results of the effect of environment on the value of the averaged CVLL using ANOVA or non-parametric equivalent. Underneath are displayed the results of post-hoc comparisons between each environment (if applicable). P-values are adjusted with Bonferroni corrections and significative differences (*p* <.05) are highlighted in bold.
