# Supplementary material for: Balance between breadth and depth in human many-alternative decisions
Source: eLife. 2022 Sep 15;11:e76985. doi: 10.7554/eLife.76985 (PMC9578699; doi:10.7554/eLife.76985)
Supplement: Supplementary file 7. [file elife-76985-supp7.docx]

|  | pure breadth | sqrt | optimal | linear | power |
| --- | --- | --- | --- | --- | --- |
| depth | $\boldsymbol{V}_{\boldsymbol{126}}\boldsymbol{=12619,}$  $\boldsymbol{p}_{\boldsymbol{adj}}\boldsymbol{=.009}$ | $\boldsymbol{V}_{\boldsymbol{126}}\boldsymbol{=19671,}$  $\boldsymbol{p}_{\boldsymbol{adj}}\boldsymbol{=7.29}\boldsymbol{\times10}^{\boldsymbol{-33}}$ | $\boldsymbol{V}_{\boldsymbol{126}}\boldsymbol{=19177,}$  $\boldsymbol{p}_{\boldsymbol{adj}}\boldsymbol{=1.09}\boldsymbol{\times10}^{\boldsymbol{-29}}$ | $\boldsymbol{V}_{\boldsymbol{126}}\boldsymbol{=19480,}$  $\boldsymbol{p}_{\boldsymbol{adj}}\boldsymbol{=1.28}\boldsymbol{\times10}^{\boldsymbol{-31}}$ | $\boldsymbol{V}_{\boldsymbol{126}}\boldsymbol{=19691,}$  $\boldsymbol{p}_{\boldsymbol{adj}}\boldsymbol{=5.37}\boldsymbol{\times10}^{\boldsymbol{-33}}$ |
| pure breadth |  | $\boldsymbol{V}_{\boldsymbol{126}}\boldsymbol{=12519,}$  $\boldsymbol{p}_{\boldsymbol{adj}}\boldsymbol{=.014}$ | $\boldsymbol{V}_{\boldsymbol{126}}\boldsymbol{=16912,}$  $\boldsymbol{p}_{\boldsymbol{adj}}\boldsymbol{=3.32}\boldsymbol{\times10}^{\boldsymbol{-17}}$ | $\boldsymbol{V}_{\boldsymbol{126}}\boldsymbol{=19681,}$  $\boldsymbol{p}_{\boldsymbol{adj}}\boldsymbol{=6.26}\boldsymbol{\times10}^{\boldsymbol{-33}}$ | $\boldsymbol{V}_{\boldsymbol{126}}\boldsymbol{=19673,}$  $\boldsymbol{p}_{\boldsymbol{adj}}\boldsymbol{=7.06}\boldsymbol{\times10}^{\boldsymbol{-33}}$ |
| sqrt |  |  | $\boldsymbol{V}_{\boldsymbol{126}}\boldsymbol{=16429,}$  $\boldsymbol{p}_{\boldsymbol{adj}}\boldsymbol{=5.55}\boldsymbol{\times10}^{\boldsymbol{-15}}$ | $\boldsymbol{V}_{\boldsymbol{126}}\boldsymbol{=17505,}$  $\boldsymbol{p}_{\boldsymbol{adj}}\boldsymbol{=3.80}\boldsymbol{\times10}^{\boldsymbol{-20}}$ | $\boldsymbol{V}_{\boldsymbol{126}}\boldsymbol{=19560,}$  $\boldsymbol{p}_{\boldsymbol{adj}}\boldsymbol{=3.88}\boldsymbol{\times10}^{\boldsymbol{-32}}$ |
| optimal |  |  |  | $\boldsymbol{V}_{\boldsymbol{126}}\boldsymbol{=15310,}$  $\boldsymbol{p}_{\boldsymbol{adj}}\boldsymbol{=2.04}\boldsymbol{\times10}^{\boldsymbol{-10}}$ | $\boldsymbol{V}_{\boldsymbol{126}}\boldsymbol{=19295,}$  $\boldsymbol{p}_{\boldsymbol{adj}}\boldsymbol{=1.96}\boldsymbol{\times10}^{\boldsymbol{-30}}$ |
| linear |  |  |  |  | $\boldsymbol{V}_{\boldsymbol{126}}\boldsymbol{=16459,}$  $\boldsymbol{p}_{\boldsymbol{adj}}\boldsymbol{=6.61}\boldsymbol{\times10}^{\boldsymbol{-27}}$ |

***Table S7***. Summary of the pair-wise comparisons (Wilcoxon Matched Pairs Signed-Ranks test) of the individual AIC between all six models using Binomial distributed noise. P-values are adjusted with Bonferroni corrections and significative differences (*p* <.05) are highlighted in bold. Models are ordered from worst (depth) to best (power).
