# Supplementary material for: Balance between breadth and depth in human many-alternative decisions
Source: eLife. 2022 Sep 15;11:e76985. doi: 10.7554/eLife.76985 (PMC9578699; doi:10.7554/eLife.76985)
Supplement: Supplementary file 9. [file elife-76985-supp9.docx]

|  | pure breadth | depth | optimal | linear | power |
| --- | --- | --- | --- | --- | --- |
| sqrt | $V_{126}=9657,$  $p_{adj}=1$ | $\boldsymbol{V}_{\boldsymbol{126}}\boldsymbol{=12670,}$  $\boldsymbol{p}_{\boldsymbol{adj}}\boldsymbol{=.007}$ | $\boldsymbol{V}_{\boldsymbol{126}}\boldsymbol{=16059,}$  $\boldsymbol{p}_{\boldsymbol{adj}}\boldsymbol{=2.22}\boldsymbol{\times10}^{\boldsymbol{-13}}$ | $\boldsymbol{V}_{\boldsymbol{126}}\boldsymbol{=16878,}$  $\boldsymbol{p}_{\boldsymbol{adj}}\boldsymbol{=4.80}\boldsymbol{\times10}^{\boldsymbol{-17}}$ | $\boldsymbol{V}_{\boldsymbol{126}}\boldsymbol{=19532,}$  $\boldsymbol{p}_{\boldsymbol{adj}}\boldsymbol{=5.85}\boldsymbol{\times10}^{\boldsymbol{-32}}$ |
| pure breadth |  | $V_{126}=10829,$  $p_{adj}=1$ | $\boldsymbol{V}_{\boldsymbol{126}}\boldsymbol{=16996,}$  $\boldsymbol{p}_{\boldsymbol{adj}}\boldsymbol{=1.31}\boldsymbol{\times10}^{\boldsymbol{-17}}$ | $\boldsymbol{V}_{\boldsymbol{126}}\boldsymbol{=18221,}$  $\boldsymbol{p}_{\boldsymbol{adj}}\boldsymbol{=4.26}\boldsymbol{\times10}^{\boldsymbol{-24}}$ | $\boldsymbol{V}_{\boldsymbol{126}}\boldsymbol{=19531,}$  $\boldsymbol{p}_{\boldsymbol{adj}}\boldsymbol{=5.94}\boldsymbol{\times10}^{\boldsymbol{-32}}$ |
| depth |  |  | $\boldsymbol{V}_{\boldsymbol{126}}\boldsymbol{=16421,}$  $\boldsymbol{p}_{\boldsymbol{adj}}\boldsymbol{=6.03}\boldsymbol{\times10}^{\boldsymbol{-15}}$ | $\boldsymbol{V}_{\boldsymbol{126}}\boldsymbol{=17137,}$  $\boldsymbol{p}_{\boldsymbol{adj}}\boldsymbol{=2.78}\boldsymbol{\times10}^{\boldsymbol{-18}}$ | $\boldsymbol{V}_{\boldsymbol{126}}\boldsymbol{=19408,}$  $\boldsymbol{p}_{\boldsymbol{adj}}\boldsymbol{=3.74}\boldsymbol{\times10}^{\boldsymbol{-31}}$ |
| optimal |  |  |  | $V_{126}=9909,$  $p_{adj}=1$ | $\boldsymbol{V}_{\boldsymbol{126}}\boldsymbol{=15828,}$  $\boldsymbol{p}_{\boldsymbol{adj}}\boldsymbol{=2.00}\boldsymbol{\times10}^{\boldsymbol{-12}}$ |
| linear |  |  |  |  | $\boldsymbol{V}_{\boldsymbol{126}}\boldsymbol{=16511,}$  $\boldsymbol{p}_{\boldsymbol{adj}}\boldsymbol{=1.99}\boldsymbol{\times10}^{\boldsymbol{-29}}$ |

***Table S9***. Summary of the pair-wise comparisons (Wilcoxon Matched Pairs Signed-Ranks test) of the individual AIC between all six models using Gaussian distributed noise. P-values are adjusted with Bonferroni corrections and significative differences (*p* <.05) are highlighted in bold. Models are ordered from worst (square root) to best (power).
