# Supplementary material for: Balance between breadth and depth in human many-alternative decisions
Source: eLife. 2022 Sep 15;11:e76985. doi: 10.7554/eLife.76985 (PMC9578699; doi:10.7554/eLife.76985)
Supplement: Supplementary file 10. [file elife-76985-supp10.docx]

| Designs  tests | B10 | W10 | B32 | W32 |
| --- | --- | --- | --- | --- |
| ANOVA | $\boldsymbol{K-W \chi}_{\boldsymbol{2}}^{\boldsymbol{2}}\boldsymbol{=17.05,}$  $\boldsymbol{p=1.99}\boldsymbol{\times10}^{\boldsymbol{-4}}$ | $\boldsymbol{F}_{\boldsymbol{1}}\boldsymbol{=20.22,}$  $\boldsymbol{p=3.18}\boldsymbol{\times10}^{\boldsymbol{-4}}$ | $F_{1}=1.52,$  $p=.23$ | $\boldsymbol{F}_{\boldsymbol{1}}\boldsymbol{=11.32,}$  $\boldsymbol{p=.0037}$ |
| Poor vs. Neutral | $\boldsymbol{W}_{\boldsymbol{15}}\boldsymbol{=206,}$  $\boldsymbol{p}_{\boldsymbol{adj}}\boldsymbol{=3.00}\boldsymbol{\times10}^{\boldsymbol{-4}}$ | $\boldsymbol{t}_{\boldsymbol{17}}\boldsymbol{=3.48,}$  $\boldsymbol{p}_{\boldsymbol{adj}}\boldsymbol{=.009}$ | - | $t_{17}=2.08,$  $p_{adj}=.16$ |
| Poor vs. Rich | $\boldsymbol{W}_{\boldsymbol{15}}\boldsymbol{=190,}$  $\boldsymbol{p}_{\boldsymbol{adj}}\boldsymbol{=.004}$ | $\boldsymbol{t}_{\boldsymbol{17}}\boldsymbol{=4.50,}$  $\boldsymbol{p}_{\boldsymbol{adj}}\boldsymbol{=9.54}\boldsymbol{\times10}^{\boldsymbol{-4}}$ | - | $\boldsymbol{t}_{\boldsymbol{17}}\boldsymbol{=3.36,}$  $\boldsymbol{p}_{\boldsymbol{adj}}\boldsymbol{=.011}$ |
| Neutral vs. Rich | $W_{15}=123,$  $p_{adj}=1$ | $t_{17}=0.362,$  $p_{adj}=1$ | - | $t_{17}=2.29,$  $p_{adj}=.11$ |

***Table S10***. Summary of the comparisons between the individual AIC in each environment and experimental design using Gaussian distributed noise. In the first row are displays results of the effect of environment on the value of AIC using ANOVA or non-parametric equivalent. Underneath are displayed the results of post-hoc comparisons between each environment (if applicable). P-values are adjusted with Bonferroni corrections and significative differences (*p* <.05) are highlighted in bold.
